# Effect of Protein Corona on The Transfection Efficiency of Lipid-Coated Graphene Oxide-Based Cell Transfection Reagents

**DOI:** 10.3390/pharmaceutics12020113

**Published:** 2020-01-30

**Authors:** Erica Quagliarini, Riccardo Di Santo, Sara Palchetti, Gianmarco Ferri, Francesco Cardarelli, Daniela Pozzi, Giulio Caracciolo

**Affiliations:** 1 Department of Chemistry, Sapienza University of Rome, P.le A. Moro 5, 00185 Rome, Italy; erica.quagliarini@uniroma1.it; 2 Department of Molecular Medicine, Sapienza University of Rome, Viale Regina Elena 291, 00161 Rome, Italy; riccardo.disanto@uniroma1.it (R.D.S.); sara.palchetti@uniroma1.it (S.P.); 3 Laboratorio NEST, Scuola Normale Superiore, Piazza San Silvestro 12, 56127 Pisa, Italy; gianmarco.ferri@sns.it (G.F.); francesco.cardarelli@sns.it (F.C.)

**Keywords:** graphene oxide, cationic lipids, gene delivery, protein corona, bionano interactions

## Abstract

Coating graphene oxide nanoflakes with cationic lipids leads to highly homogeneous nanoparticles (GOCL NPs) with optimised physicochemical properties for gene delivery applications. In view of in vivo applications, here we use dynamic light scattering, micro-electrophoresis and one-dimensional sodium dodecyl sulfate polyacrylamide gel electrophoresis to explore the bionano interactions between GOCL/DNA complexes (hereafter referred to as ”grapholipoplexes”) and human plasma. When exposed to increasing protein concentrations, grapholipoplexes get covered by a protein corona that evolves with protein concentration, leading to biocoronated complexes with modified physicochemical properties. Here, we show that the formation of a protein corona dramatically changes the interactions of grapholipoplexes with four cancer cell lines: two breast cancer cell lines (MDA-MB and MCF-7 cells), a malignant glioma cell line (U-87 MG) and an epithelial colorectal adenocarcinoma cell line (CACO-2). Luciferase assay clearly indicates a monotonous reduction of the transfection efficiency of biocoronated grapholipoplexes as a function of protein concentration. Finally, we report evidence that a protein corona formed at high protein concentrations (as those present in in vivo studies) promotes a higher capture of biocoronated grapholipoplexes within degradative intracellular compartments (e.g., lysosomes), with respect to their pristine counterparts. On the other hand, coronas formed at low protein concentrations (human plasma = 2.5%) lead to high transfection efficiency with no appreciable cytotoxicity. We conclude with a critical assessment of relevant perspectives for the development of novel biocoronated gene delivery systems.

## 1. Introduction

In the last years, the interest in manifold nanomaterials has rapidly been growing. Among these, graphene oxide (GO) is particularly promising as a potential nano-vector for several biomedical applications because of its peculiar physicochemical surface properties (e.g., high dispersibility in aqueous solvents, high biocompatibility and high specific surface area) [1,2,3,4]. Taking advantage of microfluidics, we have recently developed a hybrid nanoparticle (NP) system made of GO nanoflakes coated with cationic lipids (CL) (GOCL NPs) [5]. When administered to human cervical cancer cells (Hela), GOCL/DNA complexes (henceforward referred to as “grapholipoplexes”) were found to be as efficient as Lipofectamine^®^ 3000, the gold standard of lipid-based transfection, but definitely less cytotoxic [6]. However, when gene delivery systems are designed for in vivo applications, a critical factor is frequently ignored: the bionano interactions with biomolecules after their entry into physiological environments [7]. In fact, upon exposure to biological fluids such as human plasma (HP), nanomaterials get covered by a layer of biomolecules around their surface [8,9,10]. Due to its peculiar protein enrichment, this biomolecular envelope is frequently referred to as protein corona (PC) [11]. A PC alters the surface conformation and physicochemical properties of the pristine nanomaterials (i.e., their “synthetic identity”), thus, shaping a new “biological identity” that ultimately leads to specific physiological response [12,13]. As a matter of fact, the corona represents the molecular identity that is firstly seen (and processed) by living cells [14]. Factors shaping a PC, such as the NPs’ properties (e.g., surface area, surface charge, shape and solubility); protein source (e.g., plasma vs. serum) and environmental influences (e.g., incubation temperature, exposure time and shear stress) have all been largely documented [15,16,17,18,19]. Among these factors, the amount of proteins in the physiological environment may significantly affect the tissue engineering (TE) of gene delivery systems [14]. For instance, Dawson and co-workers have clarified that NP-protein complexes that are studied in vitro, where the protein concentration is low, may show weak correlation to their counterpart in vivo, where protein concentration is much higher [20]. More recently, it has been shown that NP-cell interactions are strongly influenced by protein concentration [17]. We have recently understood that, depending on the route of administration (e.g., systemic vs. administration), PC of gene delivery systems can change in response to bodily fluids with completely different protein concentrations (e.g., gastrointestinal fluid [21] vs. lung surfactant [22], etc.). For all these reasons, exploring the bionano interactions with the biological milieu is emerging as the missing link between benchtop discoveries and clinical applicability of gene delivery systems [7]. 

In light of such preliminary knowledge, here we investigate the bionano interactions between grapholipoplexes and HP and provide insight into the transfection behaviour of biocoronated grapholipoplexes. TE and cytotoxicity of biocoronated grapholipoplexes are assessed in four cancer cell lines: two breast cancer cell lines (MDA-MB and MCF-7 cells), a glioblastoma cell line (U-87 MG) and a colorectal adenocarcinoma cell line (CACO-2). Finally, to provide a mechanistic explanation of biological performances of biocoronated grapholipoplexes, a fluorescence confocal microscopy analysis is used.

## 2. Materials and Methods

### 2.1. Chemicals

Cationic lipids (3β-[*N*-(*N*’,*N*’-dimethyl-aminoethane)-carbamoyl])-cholesterol (DC-Chol) and 1,2-Dioleoyl-3-trimethyl-ammonium-propane (DOTAP) were purchased from Avanti Polar Lipids (Alabaster, AL). Cholesterol (Chol) was purchased from Sigma-Aldrich, Inc. (Merk KGaA, Darmstadt, Germany). Lipids were used without additional modification. Graphene Oxide (GO) aqueous dispersion was acquired by Graphenea (Donostia, Spain). Human plasma (HP) was purchased from Sigma-Aldrich, Inc. (Merk KGaA, Darmstadt, Germany). The transfected DNA (pmirGLO plasmid vector) was purchased from Promega Corporation (Madison, WI, USA). Cy3-labelled plasmid DNA (Label IT Plasmid Delivery Control Cy3) was purchased from Mirus Bio (Madison, WI, USA). Lysotracker (LysotrackerTM Deep Red, 1 mM in DMSO) was purchased from Thermo Fisher. DAPI (Prolong Diamond Antifade mountant with DAPI) was purchased from Life Technologies (Carlsbad, CA, USA). 

### 2.2. Preparation of GOCL Nanoparticles

A GO solution (1 mg·mL^−1^) was subjected three times to a pulsed sonication (2 h, 125 W per time). After each sonication, the solution was centrifuged for 10 min at 10,000 RCF (Centrifuge Z 216 MK, Hermle, Labortechnik, Wehingen, Germany), and the supernatant was recovered for the next experiment. Lastly, GO concentration was determined by spectrophotometric measurements (V-630 UV–VIS Spectrophotometer, JASCO, Deutschland). As explained in our last work, the desired lipid formulation was obtained by combining a proper percentage of DOTAP, DC-Chol and Chol (1:1:2 molar ratio, respectively). The NanoAssemblr™ Benchtop (Precision Nanosystems, Inc., Vancuver, BC, Canada) was used to obtain a homogenous solution of GO-cationic lipid (GOCL) nanoparticles (NPs). The NanoAssemblr™ Benchtop employs a microfluidic cartridge (NanoAssemblr™ Benchtop Cartridges) and pumps two different solutions into two microfluidic channels that converge in a terminal channel by fine-tuning mixing parameters as Total Flow Rate (TFR) and Flow Rate Ratio (FRR). Thanks to this experimental setup, 3 mL of GO water solution (0.2 mg·mL^−1^), prepared as described above, and 3 mL of the lipid formulation (1.5 mg·mL^−1^), dissolved in ethanol, were mixed (TFR = 12 mL·min^−1^; FRR = 1:1). The final solution was dialysed using Slide-A-Lyzer™ Cassettes (Thermo Fisher Scientific, Waltham, MA, USA) to remove the ethanol and obtain a water solution of GOCL NPs (0.1 mg·mL^−1^; referred to as GO concentration). 

### 2.3. Preparation of Grapholipoplexes

Grapholipoplexes were prepared by incubating (20 min at room temperature) 1 µg of plasmid DNA with GOCL NPs at different DNA/GOCL weight ratios (R_W_), i.e., R_W_ = 0, 0.1, 0.2, 0.3, 0.4, 0.85 and 1.6. A biophysical characterisation of the complexes was performed by dynamic light scattering (DLS) and microelectrophoresis experiments. 

### 2.4. Preparation of Grapholipopex-Protein Complexes

Human plasma (HP) was purchased from Sigma-Aldrich, Inc. GOCL/DNA-protein complexes were prepared by incubating (1 h, 37 °C) a GOCL/DNA solution (R_W_ = 0.2) at different protein concentrations: HP = 1%, 2.5%, 5%, 10%, 20% and 50% (vol/vol). A biophysical characterisation of the complexes was performed by DLS, microelectrophoresis and sodium dodecyl sulphate polyacrylamide gel electrophoresis (SDS-PAGE) experiments.

### 2.5. Size and Zeta–Potential Measurements

A Malvern NanoZetaSizer spectrometer (Malvern, Herrenberg, Germany), equipped with a digital logarithmic correlator and a 5 mW He–Ne laser (λ = 632.8 nm), was used for size and zeta-potential experiments. The Stokes Einstein equation was used to estimate the hydrodynamic radius (RH) of NPs [23,24]. Zeta potential was calculated by the Smoluchowski equation [25]. Malvern micro cuvettes (ZEN0040) were used for size measurements and a Dip Cell Kit (ZEN1002) for zeta-potential ones. Each experiment was performed at fixed temperature (25 °C) with automatic attenuator. For each sample, three independent measurements were averaged. 

### 2.6. Cell Culture

Breast cancer cell lines (MDA-MB and MCF-7 cells), glioblastoma cell line (U-87 MG) and colorectal adenocarcinoma cell line (CACO-2) were acquired from ATCC (Manassas, VA, USA). MDA-MB, MCF-7 and U-87 cells were preserved in Dulbecco’s Modified Eagle Medium (DMEM) supplemented with 10% fetal bovine serum (FBS). CACO-2 cells were preserved in Roswell Park Memorial Institute (RPMI) medium supplemented with 20% FBS.

### 2.7. Transfection Efficiency Experiments

MDA-MB, MCF-7, U-87 MG and CACO-2 cells were seeded on a 96-well plates (10,000 cells/well). Each cell line was treated with GOCL/DNA-protein complexes in Optimem medium (Life Technologies, Carlsbad, CA, USA) for 3 h and then the medium was replaced with DMEM 10% FBS for MDA-MB, MCF-7 and U-87 MG and RPMI 20% FBS for CACO-2. After 48 h, cells were analysed for luciferase expression by means of Luciferase Assay System (Promega, Madison, WI, USA). Briefly, cells were washed with phosphate saline buffer (PBS) and collected with 20 μL 1X Lysis buffer (Promega), then 10 μL of the cell suspension was diluted with 100 μL luciferase substrate (Promega) and the remaining 10 uL was used for BCA assay. The transfection efficiency expressed was determined by Pierce BCA Assay Protein Kit (Thermo Fisher Scientific, Waltham, MA, USA) and expressed as Relative Light Units (RLU) per mg of cell proteins.

### 2.8. Cell Viability Experiments

To determine the cytotoxicity arising from the administration of GOCL-pDNA-protein complexes, cell viability of MDA-MB, MCF-7, U-87 MG and CACO-2 cell lines were evaluated by 3-(4,5-dymethyl thiazol 2-y1)-2,5-diphenyl tetrazolium bromide assay (MTT assay, mitochondrial respiration analysis; Sigma-Aldrich), according to Mosmann protocol. In brief, cells were seeded on 96-well plates (10,000 cells/well). The day after, cells were incubated with GOCL-pDNA-protein complexes in Optimem medium. After 3 h, the medium was replaced with DMEM 10% FBS for MDA-MB, MCF-7 and U-87 MG and RPMI 20% FBS for CACO-2, and the cells were incubated for 48 h at 37 °C. Thus, MTT was added to each well at the final concentration of 0.5 mg/mL and, after 3 h of incubation at 37 °C, the formazan salt was dissolved with 100 µL of isopropyl alcohol. The absorbance of each well was measured with Glomax Discover System (Promega, Madison, WI, USA), a ready-to-use high-performance multimode detection instrument.

### 2.9. 1D SDS PAGE Gel Electrophoresis

For the protein corona characterisation, 1D SDS PAGE gel electrophoresis was performed. GOCL-protein complexes with and without pDNA were centrifuged three times for 15 min at 18,620 RCF at 4 °C, and the pellets were washed, each time, with ultrapure water to remove unbound proteins. After the washes, the pellets were suspended in 20 µL of Laemmli loading buffer 1x, boiled for 10 min at 100 °C and centrifuged at 18,620 RCF for 15 min at 4 °C. Supernatants were collected and loaded on a stain-free gradient polyacrylamide gel (4–20% TGX precast gels, Bio-Rad, Hercules, CA, USA) and run at 100 V for about 150 min. Finally, gel images were acquired with a ChemiDoc™ gel imaging system (Bio-Rad, CA, USA) and processed by means of custom MatLab scripts (MathWorks, Natick, MA, USA) [26]. 

### 2.10. Confocal Microscopy

MDA-MB, MCF-7, U-87 MG and CACO-2 cells were seeded onto 12 mm round glass coverslips (150.000 cells/coverslip). After 24 h, a GOCL solution was incubated with 1 µg of Cy3-labeled plasmid DNA (Label IT Plasmid Delivery Control Cy3, Mirus Bio, USA) (R_W_ = 0.2) for 20′ at RT and next with HP (1 h at 37 °C) at different concentrations, i.e., 1%, 2.5%, 5%, 10%, 20% and 50% (vol/vol). The GOCL/DNA-protein complexes were treated with 1 mL of a solution 1:2000 Optimem/Lysotracker (LysotrackerTM Deep Red, 1 mM in DMSO, Thermo Fisher), and then each sample was administered to cells. After 3 h, cells were fixated with 500 μL of p-Formaldehyde 4% for 10′ and treated with DAPI (Prolong Diamond Antifade mountant with DAPI, Life technologies, Carlsbad, CA, USA). Fluorescence imaging experiments were performed using a Zeiss LSM 880 AiryScan confocal microscope with a 63X 1.4 N.A. oil immersion objective and GaAsP detectors. Imaging of the three different fluorescent species present in the sample was carried out in sequential mode to avoid cross-talk between emission spectra. DNA-Cy3 was excited at 561 nm and emission collected in the 545–645 nm range, DAPI was excited at 405 nm and emission collected in the 410–545 nm range and LysoTracker Deep Red was excited at 640 nm and emission collected in 645–700 nm range. Each image consists of 1024 × 1024 pixels with a pixel size of 60 nm. 

### 2.11. Statistical Analysis

The results are reported as mean ± S.D. and are represented as error bars in the graphs. For each sample, *n* = 3 independent measurements were averaged. Statistical significance was evaluated using Student’s *t*-test (* *p* < 0.05; ** *p* < 0.01) with respect to pristine grapholipoplexes (i.e., 0% HP) and untreated cells in TE and cell viability experiments, respectively. 

## 3. Results

### 3.1. Size and Zeta-Potential of Grapholipoplexes

**Figure 1:**
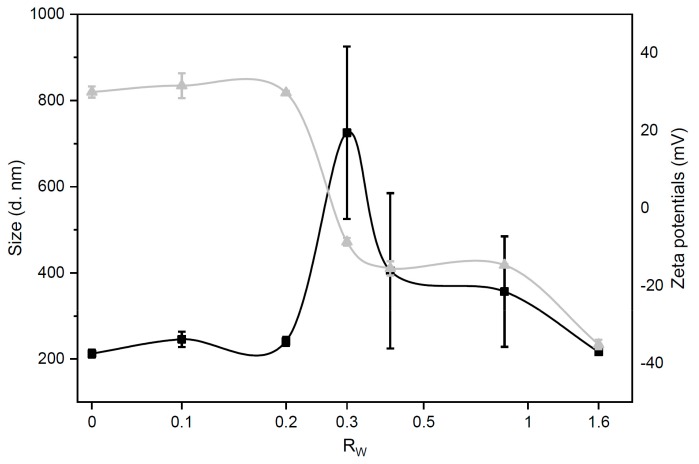
Size (black squares) and zeta-potential (grey triangles) of grapholipoplexes as a function of the DNA/ graphene oxide nanoflakes with cationic lipids (GOCL) weight ratio, R_W_. Grapholipoplexes exhibit typical charge inversion and “re-entrant condensation”. At the iso-electric point (R_W_ = 0.3), grapholipoplexes are neutrally charged and large in size (~800 nm). This is due to short-range van der Waals attraction that prevails over electrostatic repulsion between positively charged GOCL nanoparticles (NPs) and negatively charged DNA. On both sides of the evolution profile, grapholipoplexes are small-sized (~200 nm), with surface charge dominated by excess DNA (R_W_ > 0.3) or excess cationic lipid (R_W_ < 0.3).

NP concentration is a key factor in cellular experiments, as it is directly correlated to the sample administration volume. Indeed, administration of large sample volumes have been reported to negatively interfere with cellular survival, challenging the osmotic equilibrium of the cell membrane [27]. With respect to previous investigations [1,5], here GOCL NPs were prepared at higher concentrations that allowed us to use a lower administration sample volume, thus, minimising unwanted effects on cell cultures. Since concentration may affect the aggregation state of colloidal systems, a preliminary characterisation was performed to evaluate size and zeta-potential of GOCL NPs. DLS and microelectrophoresis measurements showed that GOCL NPs had optimal physicochemical properties for gene delivery purposes, as they were small in size (212.3 ± 9.1 nm, diameter) and positively charged (zeta-potential = 30.0 ± 1.5 mV) (data not reported for space consideration). It is well-known that the DNA/CL weight ratio (R_w_) is a key factor affecting CL-based gene delivery [28,29]. As a consequence, we first characterised size and zeta-potential of grapholipoplexes as a function of R_w_. Figure 1 shows that grapholipoplexes undergo re-entrant condensation and charge inversion [30]. 

At the isoelectric point (R_w_ = 0.3), electrostatic balance between positively charged GOCL NPs and negatively charged DNA gives rise to formation of large-size (725.2 ± 200.4 nm) and weakly charged (zeta-potential = −8.7 ± 0.9 mV) grapholipoplexes. This is a consequence of short-range van der Waals interactions that dominate over electrostatic repulsions between single-DNA-decorated particles [30]. Overloading GOCL NPs with excess DNA results in a diminution of both size and zeta potential of complexes. At R_w_ = 1.6, grapholipoplexes are small in size (216.3 ± 7.0 nm) and negatively charged (zeta-potential = −35.1 ± 1.2 mV). On the other side, decreasing DNA content with respect to isoelectric point leads to small-sized, positively charged complexes. It is well-known that cationic gene delivery systems are more efficient in transfecting mammalian cells than anionic ones [31]. This depends on favourable interactions between positively charged gene delivery systems and negatively charged cell proteoglycans. As a consequence, cationic grapholipoplexes (R_w_ = 0.2) were used in the following experiments.

### 3.2. Size and Zeta-Potential of Biocoronated Grapholipoplexes

**Figure 2:**
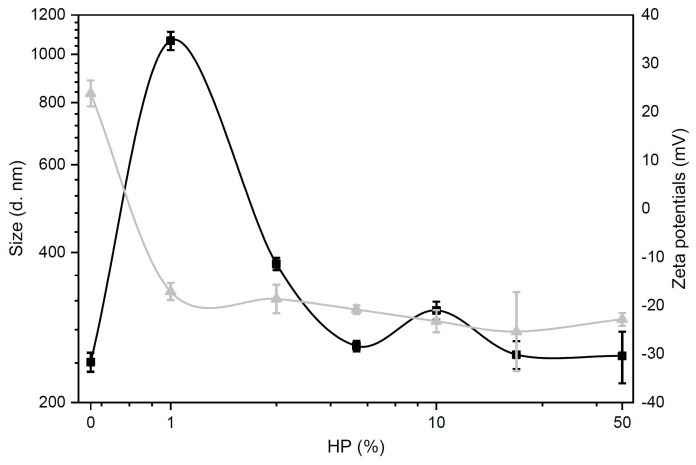
Size (black squares) and zeta-potential (grey triangles) of biocoronated grapholipoplexes as a function of human plasma (HP) concentration.

Following exposure to biological media, the physicochemical properties of NPs are largely altered [15]. Thus, we explored the effect of protein concentration on size and zeta-potential of grapholipoplexes. In accordance with previous literature [32], biocoronated grapholipoplexes showed a significant increase in size and a rapid inversion of zeta-potential from positive to negative values (Figure 2). Since plasma proteins are mainly anionic at physiological pH, already with 1% HP, the cationic surface charge of grapholipoplexes is rapidly reversed to negative values (zeta-potential = −17.1 ± 1.8 mV). With the increasing HP concentration, zeta-potential remains at net negative values with minor fluctuations, indicating that complexes are completely covered by plasma proteins and that a further increase of HP concentration does not lead to a further thickening of the corona [17]. A characteristic pattern for size of coronated grapholipoplexes was also observed. At HP = 1%, complexes are large in size, meaning that a rapid particle clustering has occurred as a result of charge neutrality [30]. A further increase of HP concentration results in marked size decrease, until a plateau is reached at around 5% HP exposure.

### 3.3. Protein Corona of Grapholipoplexes

**Figure 3:**
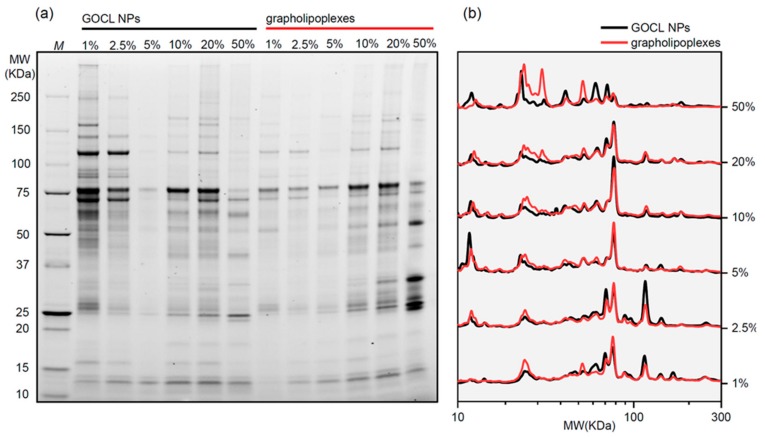
Protein corona formed around GOCL NPs and grapholipoplexes (R_w_ = 0.2), following 1-h exposure to human plasma at 37 °C. (**a**) 1D SDS-PAGE gel of corona proteins obtained from GOCL NPs and grapholipoplexes upon incubation with different amount of HP (i.e., 1%, 2.5%, 5%, 10%, 20% and 50%, vol/vol). The molecular weight (MW) of the proteins indicated near standard ladder (*M*). (**b**) Comparison of 1D protein profiles of coronas formed around GOCL NPs (black curves) and grapholipoplexes (red curves).

Coronas of GOCL NPs and grapholipoplexes were characterised through 1D SDS-PAGE (a representative gel image is shown in Figure 3a). First, we observed that PC composition of both systems was influenced by protein concentration. To compare coronas of GOCL NPs and grapholipoplexes, 1D normalised protein patterns were calculated (Figure 3b). At low protein concentration (HP < 5%), protein patterns of GOCL NPs and grapholipoplexes were almost superimposable. On the other side, at high protein concentration (HP < 20%), marked differences in the protein profiles of GOCL NPs and grapholipoplexes were found. Bearing in mind that plasma proteins are largely negatively charged at physiological pH, our findings suggest that, at low protein concentration, they preferentially bind to the lipid surface of grapholipoplexes. Subsequently, once the lipid surface of grapholipoplexes is fully saturated by protein, less-abundant acidic plasma proteins bind to negatively charged plasmids located at the particle surface [33].

### 3.4. Transfection Efficiency and Cytotoxicity of Biocorononated Grapholipoplexes

**Figure 4:**
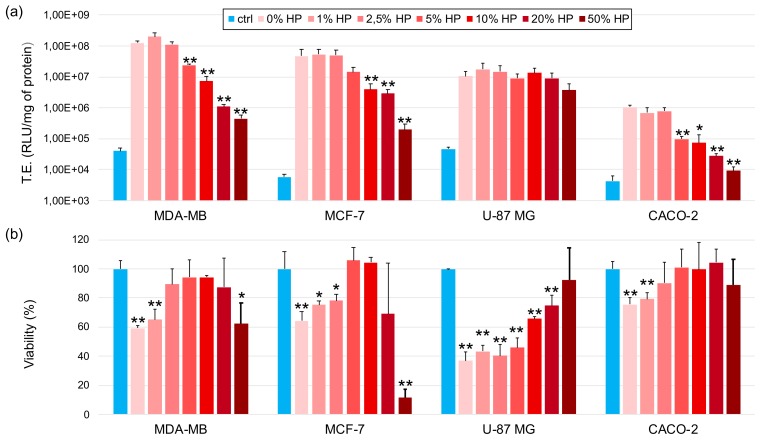
Transfection efficiency (TE, panel **a**) and cell viability (panel **b**) of biocoronated grapholipoplexes at different plasma concentrations in MDA-MB, MCF-7, U-87 MG and CACO-2 cells. Untreated cells (blue bars) were used as a control (ctrl) in both experiments. Results are given as average of six independent measurements ± S.D. Statistical significance was evaluated using Student’s *t*-test (* *p* < 0.05; ** *p* < 0.01; no asterisk means lack of significance) with respect to pristine grapholipoplexes (i.e., 0% HP) and untreated cells in TE and cell viability experiments, respectively.

Next, we explored the effect of PC on the ability of grapholipoplexes to transfect cancer cells (Figure 4a). PC had minor effect, if any, on TE of biocoronated grapholipoplexes in glioblastoma U-87 MG cells. On the other side, when biocoronated grapholipoplexes were given to MDA-MB, MCF-7 and CACO-2 cells, TE exhibited decreasing profile with increasing protein concentration. Viability of cells treated with grapholipoplexes was also assessed (Figure 4b). For both breast cancer cell lines (i.e., MDA-MB and MCF-7 cells), a nonmonotonic trend was found. The administration of pristine grapholipoplexes (i.e., in the absence of PC) reduced cell viability of MDA-MB and MCF-7 cell viability up to 59.3% ± 1.5 and 64.3% ± 6, respectively. On the other side, biocoronated grapholipoplexes increased cell viability up to HP = 10% (vol/vol) (cell viability equal to 94.3% ± 1.2 and 104.3% ± 3.2 for MDA-MB cells and MCF-7 cells, respectively). A further increase in protein concentration (i.e., HP = 20% and 50%) led to diminution on cell viability up to 62.6% ± 13.8 and 11.6% ± 5.8 for MDA-MB cells and MCF-7 cells, respectively. Cell viability of U-87 MG and CACO-2 cells regularly increased with HP concentration. Previous investigations showed that, depending on its composition, PC can have either detrimental [34,35] or protective effect on cell viability [36,37]. For instance, previous works showed that when a protein is adsorbed on NP surface, it may undergo into denaturation and trigger a cytotoxic mechanism by exposing immunogenic epitopes [38,39]. On the other side, PC can have a stealth effect on NP uptake by immune cells [40]. Available literature indicates that activation of tumour necrosis factor-related apoptosis-inducing ligand can promote apoptosis of cancer cells [41]. Our results are likely to indicate that the interplay between PC composition and cancer cell receptor profiles can affect particle-cell association [42,43] and apoptosis-inducing ligand signalling [44]. 

### 3.5. Intracellular Final Fate of Biocoronated Grapholipoplexes

It has been demonstrated the formation of PC provides a capacity to alter both cellular and intracellular location of nanoparticles [45,46,47]. Among intracellular final destinations, lysosomes are an undesirable option. Indeed, degradation of gene delivery systems in lysosomal compartment is one of the most relevant bottlenecks of efficient transfection [48,49]. Therefore, the final fate of fluorescently-labelled grapholipoplexes (green) was investigated next. 

**Figure 5:**
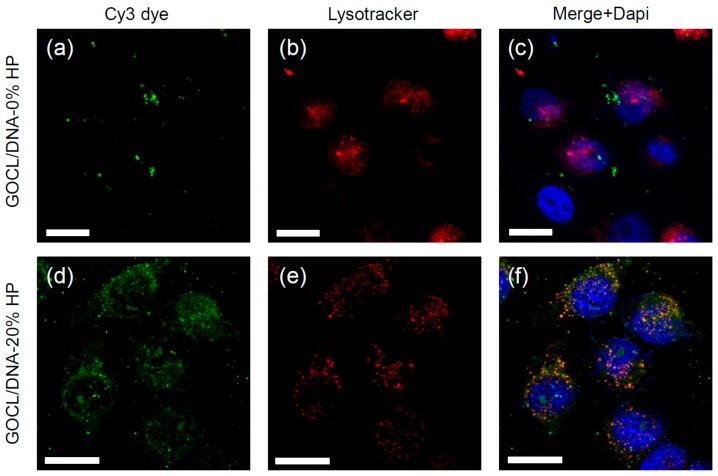
Representative confocal microscopy images of of MDA-MB cells treated with fluorescently labelled (green) pristine (panels **a**–**c**) and biocoronated grapholipoplexes (HP = 20%) (panels **d**–**f**). Cells were stained with LysoTracker Deep Red (red), a well-known lysosomal marker, and Dapi to stain the nuclei. Scale bars are 5 microns.

To this end, MDA-MB, MCF-7, U-87 MG and CACO-2 cells cancer cells were labelled with LysoTracker Deep Red (red), a well-known lysosomal marker. Grapholipoplexes colocalising with lysosomes gave rise to yellow clusters. As an example, Figure 5 compares lysosomal accumulation of pristine (Figure 5, panels a–c) and biocoronated grapholipoplexes (Figure 5, panels d–f) in MDA-MB breast cancer cells. While pristine grapholipoplexes escape lysosomal degradation (Figure 5c), their biocoronated counterpart largely accumulates within lysosomes (Figure 5f). A similar behaviour was detected in MCF-7, U-87 MG and CACO-2 cells (Figure S1). 

## 4. Discussion

Recently developed grapholipoplexes [1] have distinctive physical–chemical properties, such as small size and cationic surface charge, that make them promising candidates for gene delivery purposes. With respect to widely used cationic lipid/DNA complexes (lipoplexes) [50], grapholipoplexes are much more homogeneous in size, and, in turn, they exhibit unusual ability to split uniformly their gene payload to the whole cell population. As a consequence of their peculiar transfection behaviour, grapholipoplexes are simultaneously highly efficient and completely not cytotoxic, even at high concentrations. In view of in vivo applications, it is essential to characterise structure and biological outcomes of gene delivery systems under biologically relevant experimental conditions [10]. This work is therefore aimed at investigating the bionano interactions between grapholipoplexes and biological milieu as a function of protein concentration. First, we clarify that, following exposure to HP, grapholipoplexes get covered by a PC whose composition is affected by protein concentration. Evolution profiles of size and zeta-potential of biocoronated grapholipoplexes reported in Figure 2 closely resemble those previously reported for cationic liposome-protein complexes [51]. Considering the significant differences between grapholipoplexes and cationic liposomes in terms of shape and composition, this observation confirms that not-specific electrostatic interaction is the main driving force that regulates formation and equilibrium structure of coronated materials in biological fluids [33,52]. Results reported in Figure 3a show that PC has a detrimental effect on TE of grapholipoplexes, but such reduction in TE cannot be clearly associated to cytotoxic effects (Figure 3b). However, it is widely accepted that the final TE of gene delivery systems is rate-limited by several biological barriers beyond cytotoxicity, such as cellular uptake, endosomal escape and nuclear entry. Lessening in TE could likely correlate with surface charge properties of biocoronated grapholipoplexes. It is well-established in the literature that gene delivery systems should be positively charged to promote efficient interaction with negatively charged proteoglycans, which are an important part of the extracellular matrix. Results reported in Figure 2 show that, even at the lowest protein concentration (HP = 1%), protein binding provides biocoronated grapholipoplexes with negative zeta-potential. This inversion in surface charge is likely to be responsible for reduced electrostatic interaction with cancer cells [42] that, in turn, may lead to lower cellular uptake. In recent investigations [6], we have demonstrated that transport of DNA-loaded vesicles along microtubules frequently results in poor endosomal escape and massive lysosomal accumulation, leading to DNA degradation and poor transfection. In this view, PC formed in human plasma (e.g., upon systemic administration) may compromise endosomal escape of biocoronated grapholipoplexes, shuttling them to lysosomal compartments [47], with the result that they are much less effective than their pristine counterpart (i.e., in the absence of PC). On the other hand, the latest research has shown that pre-coating of NPs with plasma proteins allows the design of artificial coronas with controlled physiochemical properties and optimised transfection behaviours [53]. In this regard, biocoronated grapholipoplexes coated with artificial coronas formed at low protein concentration (HP < 2.5%) exhibit high TE with minor impact on cell viability. This proves that pre-coating of grapholipoplexes may be a feasible strategy to control their transfection behaviour in vivo.

## 5. Conclusions

Despite the huge amount of preclinical data involving the use of gene delivery systems, their clinical application is far from established. Recent research has revealed some overlooked factors in gene delivery, helping to elucidate the inadequate achievements to date. Among them, a lack of knowledge of the bionano interactions between nanomaterials and the physiological environment is emerging as the most significant reason why only a few nano-delivery systems have entered clinical use. In this work, we have demonstrated that the bionano interactions between plasma proteins and grapholipoplexes can have a dramatic effect on their particle identity and biological responses. Overall, our results indicate that the protein corona formed at high protein concentrations (i.e., mimetic of protein concentrations typical of in vivo studies) can reduce the transfection efficiency of grapholipoplexes in cancer cells and increase the probability that the DNA payload gets digested at the intracellular level. On the other side, pre-coating grapholipoplexes with artificial coronas formed at low protein concentrations may lead to high transfection efficiency and low cytotoxicity. In this regard, IgG-depleted plasma could be a convenient protein source to coat grapholipoplexes, as it may reduce undesired interactions with the immune system [51,54] and activate receptor-mediated endocytosis [55]. As many of us have shown, artificial coronas may remain stable upon re-exposure to biological media in vivo. A fundamental goal of future research will be understanding the molecular mechanisms that make artificial coronas of grapholipoplexes stable in the human body. This question needs to be addressed in order to engineer safe and targeted biocoronated nanomaterials. 

## Supplementary Materials

The following are available online at https://www.mdpi.com/1999-4923/12/2/113/s1: Figure S1: Merged confocal microscopy images of MCF-7, U-87 MG and CACO-2 cells treated with pristine and biocoronated grapholipoplexes incubated with HP (20%, vol/vol).
