# Supplementary material for: Effect of Protein Corona on The Transfection Efficiency of Lipid-Coated Graphene Oxide-Based Cell Transfection Reagents
Source: Pharmaceutics. 2020 Jan 30;12(2):113. doi: 10.3390/pharmaceutics12020113 (PMC7076454; doi:10.3390/pharmaceutics12020113)
Supplement: Supplementary file 1 [file pharmaceutics-12-00113-s001.pdf]

pharmaceutics

MDPI

Supplementary Materials: Effect of Protein Corona on The Transfection Efficiency of Lipid-Coated Graphene Oxide-Based Cell Transfection Reagents

Erica Quagliarini, Riccardo Di Santo, Sara Palchetti, Gianmarco Ferri, Francesco Cardarelli, Daniela Pozzi and Giulio Caracciolo

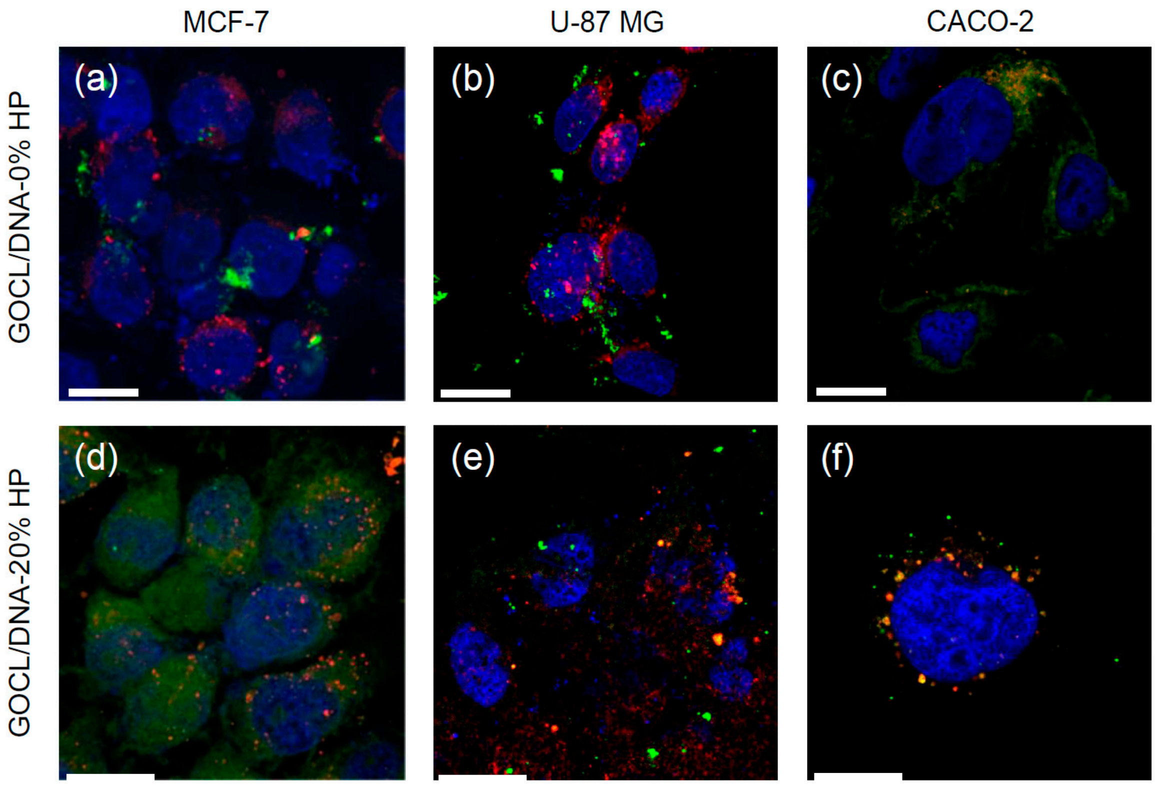

Figure S1. Merged confocal microscopy images of MCF-7, U-87 MG and CACO-2 cells treated with pristine grapholipoplexes (**a**, **b** and **c**, respectively) and grapholipoplex-protein complexes obtained by incubating pristine grapholipoplexes with human plasma (20%, vol/vol) (**d**, **e** and **f**, respectively). Grapholipoplexes were stained with Cy3-dye (green channel), lysosomes with lysotracker (red channel) and nuclei with DAPI (blue channel). Scale bars= 5 micron.
